# Energy Expenditure Estimation of Tabata by Combining Acceleration and Heart Rate

**DOI:** 10.3389/fpubh.2021.804471

**Published:** 2022-02-07

**Authors:** Yiping Yan, Qingguo Chen

**Affiliations:** Sport Laboratory, College of Physical Education, Sichuan Normal University, Sichuan, China

**Keywords:** Tabata training, machine learning, heart rate, acceleration, energy expenditure

## Abstract

Tabata training plays an important role in health promotion. Effective monitoring of exercise energy expenditure is an important basis for exercisers to adjust their physical activities to achieve exercise goals. The input of acceleration combined with heart rate data and the application of machine learning algorithm are expected to improve the accuracy of EE prediction. This study is based on acceleration and heart rate to build linear regression and back propagate neural network prediction model of Tabata energy expenditure, and compare the accuracy of the two models. Participants (*n* = 45; Mean age: 21.04 ± 2.39 years) were randomly assigned to the modeling and validation data set in a 3:1 ratio. Each participant simultaneously wore four accelerometers (dominant hand, non-dominant hand, right hip, right ankle), a heart rate band and a metabolic measurement system to complete Tabata exercise test. After obtaining the test data, the correlation of the variables is calculated and passed to linear regression and back propagate neural network algorithms to predict energy expenditure during exercise and interval period. The validation group was entered into the model to obtain the predicted value and the prediction effect was tested. Bland-Alterman test showed two models fell within the consistency interval. The mean absolute percentage error of back propagate neural network was 12.6%, and linear regression was 14.7%. Using both acceleration and heart rate for estimation of Tabata energy expenditure is effective, and the prediction effect of back propagate neural network algorithm is better than linear regression, which is more suitable for Tabata energy expenditure monitoring.

## Introduction

According to the recently published World Wide Survey of Fitness Trends, high-intensity interval training (HIIT) has become increasingly popular modes of physical exercise (1). Tabata training is one of the most energetically effective high-intensity intermittent training methods (2).This exercise is relatively inexpensive, usually requires very little equipment, and is effective in promoting physical fitness. Tabata is not only useful to promote fat loss, improve blood pressure, insulin sensitivity, and glucose regulation in a relatively short time, but also enhance sports performances that depend on both the aerobic and anaerobic energy exependiture systems (2, 3). Being able to monitor energy expenditure (EE) portable and accurately during these activities is critical for weight control, physical activity adjustment and fitness goals achievement.

At present, the methods that produce the most accurate measurement of EE is direct calorimetry, indirect calorimetry and doubly labeled water (4), however, one limitation of all three methods is that they are expensive to test, which is not suitable for mass measurement of daily physical activity (5). Both accelerometer and heart rate sensor have long been recognized as the common and cheap wearable devices used for monitoring EE (6, 7). In 2001, Strath et al. (8) proposed a method for predicting EE using accelerated combined heart rate (ACC-HR), and believed that this method is better than the single acceleration (ACC) or heart rate (HR) measurement. So far, other authors have verified that this method predict the EE of some Physical activity (PA) more accurately, such as flat running and uphill running with different intensity (9, 10). In the era of big data, machine learning (ML) has been widely used in people's production and life (11–14). In terms of EE prediction algorithm, back propagate neural network (BPNN) is one of the most common used in machine learning algorithms (15), which has been applied and has significantly improved EE prediction performance in some exercises compared with linear regression (LR).

The main contributions of this paper are:

(1) We analyzed the correlation between ACC and EE, HR and EE in Tabata training.
(2) We established linear regression and back propagate neural network model based on ACC-HR to predict the EE of Tabata training during exercise and intervals.
(3) We evaluate and compare the predictive effectiveness of linear regression and back propagate neural network model.

The rest of the paper is organized as follows. Section 2 gives literature review. Section 3 describes our proposed method in detail. Section 4 presents the test results and analysis. Section 5 discuss the result. Section 6 gives conclusions and Future work.

## Literature Review

### Existing Wearable Devices

Currently, wearable devices that monitor EE can be divided into five categories: pedometer, accelerometer, heart rate sensor and multi-sensor combination (16). Pedometer is the most convenient motion sensor, and converts the PA of exercisers through the number of walking steps. However, there are significant limitations in EE predicting for slow walking, non-walking, and running (17). The accelerometer can calculate the rate of change of speed within a certain time, and the PA parameters was obtained through body acceleration, then EE is calculated based on the relationship between the known ACC and EE. Unfortunately, the accuracy of energy monitoring is limited by different acceleration wearing positions, additional load on the user and the change of moving surface (18). Heart rate sensor was estimated based on the correlation between HR and oxygen consumption (VO_2_) of some physical activities of exercisers, and then the EE was reflected by oxygen intake. But the accuracy of HR monitoring was affected by exercise action, mood and health status (19). A potential and powerful energy expenditure prediction approach is to use ACC and HR simultaneously. ACC values confirm that increased HR is caused by physical activity, which is the basic principle behind the combination of these techniques (16). In addition, the heart rate is linear with the EE of medium and high intensity exercise, which can quantify the daily EE of most people, but it cannot quantify the low intensity physical activity. The deficiency is compensated by acceleration data, which can effectively predict low intensity physical activity (16). In this study, Tabata exercise EE was monitored, including high-intensity exercise and intermittent exercise. The ACC-HR measurements may improve the EE monitoring effect.

### Estimation Algorithm

Most of the early research and monitoring EE models are LR models, and the most classical EE linear regression model is Freedsom regression with acceleration parameters (20). Later studies confirmed that a single variable regression could not fit all physical activities, and then some studies developed linear regression models with multiple variables such as acceleration combined with body weight and heart rate combined with body weight for achieving better prediction results (10, 21). However, the ACC, HR, and EE used for some workouts are not linearly or highly correlated. Recently, Morris et al. have shown that ACC is not effective in predicting high-intensity intermittent exercise using the previous LR model (22). Recent sudden developments in the field of machine learning has increased the potential for remote monitoring and diagnostics using data obtained from wearable devices (23). Such as standing balance estimation (24), ECG signal classification (25), and EE prediction (26), etc. Some studies have achieved good accuracy by using BPNN, random forest (RF) and other machine learning algorithms to build models (23, 27). In addition, Montoye et al. (15) has proved that BPNN is better than linear regression for predicting EE in daily physical activity and partial sports. In terms of variable selection, there is inconsistency between machine learning and traditional regression model in the selection of input variables, as shown in Table 1. Recently, the researchers verified that BPNN model was constructed with ACC, HR and morphological indicators as input variables, which had high accuracy in predicting walking, running and resistance movement in EE (23). BPNN is the most popularly used Artificial neural network at present, which make up for the difficulty of linear regression in modeling non-linear data or polynomial regression with correlation between data features. For EE prediction of Tabata training, BPNN model may provide a new prediction idea. In this study, acceleration and heart rate data were selected as input variables, and the EE prediction was used by LR and BPNN model.

**Table 1:** Summary of an existing model in EE prediction.

**References**	**Predict activities**	**Model type**	**Variable selection**
Shaopeng et al. (20)	Daily physical activity, Walk, run	One-variable linear regression	Acceleration
Kuo et al. (10); Caron et al. (21)	Uphill run; walk	Mutiple linear regression	Acceleration, heart rate; morphological indicators acceleration
Montoye et al. (15)	Daily physical activity, walk, run, resistance exercise	BP neural network	Acceleration
O'Driscoll et al. (27)	Stand, walk, run, slope run	Random forest	Acceleration, heart rate, morphological indicators, subject characteristics
Kang et al. (23)	Run, cycling, resistance exercise	BP neural network	Acceleration, heart rate, body temperature

## Method

### Participants

A sample of 45 participants were included [Female = 23, Age (AG) = 21.04 ± 2.39 years, body height (BH) = 1.67 ± 0.75 m, body weight (BW) = 59.61 ± 8.27 kg, body mass index (BMI) = 21.17 ± 2.16 kg/m^2^, body fat rate (BFR) = 18.46 ± 5.47%]. Participants were primary recruited from Si Chuan Normal University. Exclusion criteria included the following: (1) Participants had a history of respiratory, cardiovascular, neurobiological, metabolic, or lower extremity related diseases, (2) Participants were given hypnotic drugs, alcohol, caffeine, or nicotine. This study was approved by the Si Chuan Normal University institutional Research Board before participant recruitment. Immediately upon arrival at the exercise research lab, the researchers described the study details to each participant and obtained written informed consent before proceeding with the protocol.

### Instrumentation

**Figure 1:**
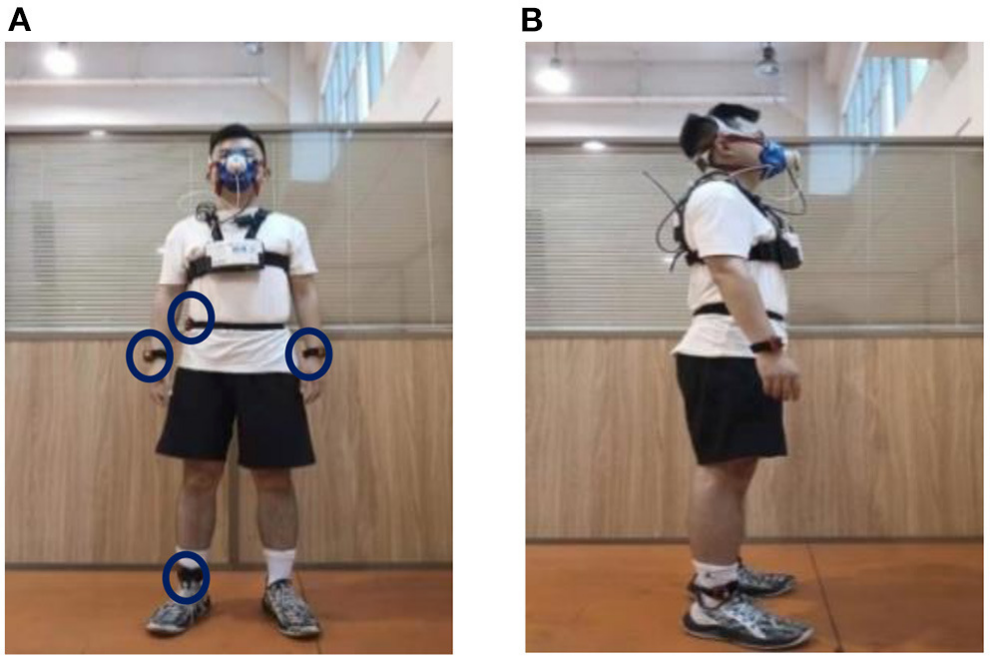
Position of instrument wearing **(A)** Front view and **(B)** Lateral view.

EE was measured by indirect calorimetry method with gas metabolism analyzer as calibration, and collects data in kilocalorie as the basic unit. During the Tabata test, participants wore a portable gas metabolism analyzer, four wear site accelerometer (dominant hand, non-dominant hand, right hip, right ankle), and a chest strap heart rate band, all set up according to the manufacturer's instructions. The portable gas metabolism analyzer (K4b2, Cosmed) was used as a calibration to measure the EE of Tabata motion, it was calibrated according to the manufacturer's instructions before test. HR was recorded by chest strap heart rate band (Polar wearlink) during exercise, because K4b2 comes with a synchronization sensor from Polar's heart rate band, the heart rate band synchronizes with K42b measurements. ACC was acquired from four accelerometer (Actigraph GT3X+), which is initialized to set the sampling frequency to 30HZ. Prior to the test, participants identified their dominant hand (defined as the hand with which they wrote), and GT3X + was placed on the dominant hand, non-dominant hand, right hip, right ankle for all tests, the instruments was worn as shown in the Figure 1. In order to ensure the synchronization of the accelerometer with k4b2 and Polar heart rate band test, calibrate the computer time of the two devices and set the acceleration start time in advance.

### Procedure

**Figure 2:**
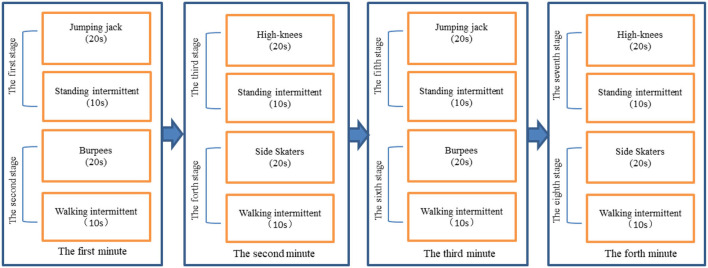
Tabata exercise process.

Each participant reported to the Sports Research Laboratory for one visit. To avoid discomfort and errors in EE measurement during the test, participants were forbidden to eat 2 h before the test. The testers explained the details of the test to each participant and obtained written informed consent. A PA preparation questionnaire was conducted prior to the test to ensure that participants were healthy and not participating in MVPA contraindications. If the participant answered “yes” to any of the questions on the PA questionnaire, they would have been asked to obtain physician approval before being able to participate in the study; however, whether there are rules in the questionnaire will not be tested. Next, trained research assistants measured height, weight, body fat percentage, and BMI using a human morphological indicator meter (InBody J30). In addition, the age shall be calculated in years according to the date of birth of the resident identity card. The classic Tabata training mode is 20–10 mode (20s-exercise, 10s-interval). In consideration of the simplicity of the exercise movements and the comprehensiveness of the exercise parts, this study adopts the classic Tabata training mode with multiple movement combinations. Participants were asked to learn how to perform the Tabata movements (Jumping jack, Burpees, Running in place high-knees, Side Skaters) and complete each movement 5 times after learning to get familiar with the movements, the exercise process is shown in Figure 2, eight movement stages were tested, each consisting of a exercise and an interval. These exercise movements not only can be full-body exercise and follow the principle of simplicity, but also have been proved by studies to achieve high intensity (28), and the intermittent movements are also common. After getting familiar with the action, participants warmed up with a 5-min warm-up in the Keep APP. Once the participant completes warm-up, Polar wearlink, Cosmed K4b2, and Actigraph GT3X fitted to the participant. Sit still for at least 15 min until heart rate stabilizes. At the beginning of the test, participants performed the maximum number of reps prompted by a Tabata video played on a computer. Relax and stretch after completing the test.

### Data Processing

#### Tabata Exercise Intensity Monitoring

**Figure 3:**
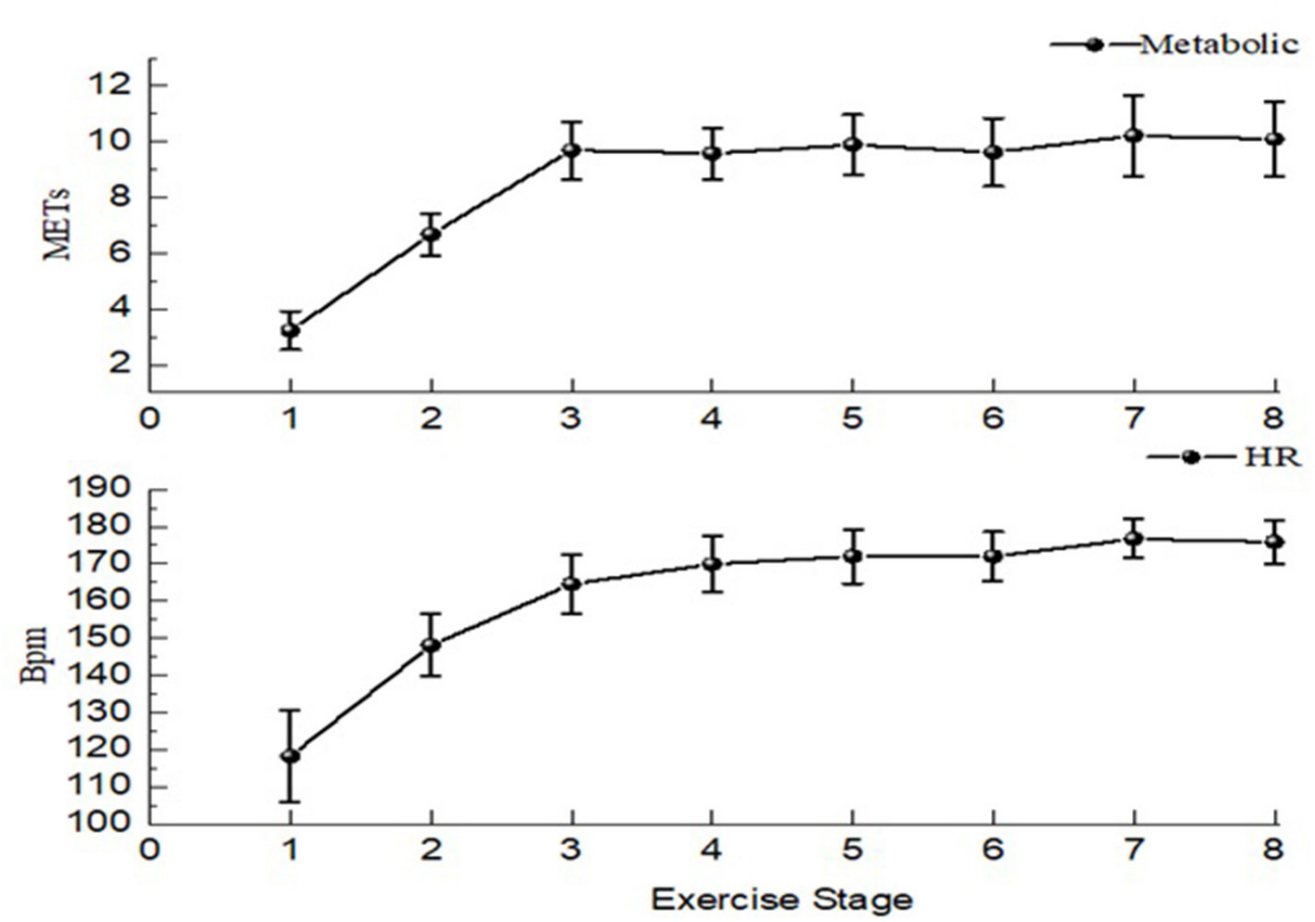
Exercise intensity test.

To ensure that participants reach the high intensity during the Tabata exercise test, the heart rate of participants and metabolic equivalent (METs) during each stage (exercise + interval) were collected during the test (Figure 3). In this study, the average HR of exercise was 165.46 ± 5.39 for men and 163.88 ± 6.88 for women, reaching 80% of the maximum heart rate. As the same time, the average of METs was 8.84 ± 0.89 for men and 8.39 ± 0.93 for women. Refer to ACSM for exercise categories and physical activity intensity (29), the exercise intensity of this test reached high intensity, Maximum heart rate 183.43 ± 7.09, almost 90% of maximum heart rate, which was in line with the exercise intensity required by Tabata exercise.

#### Variable Selection

The triaxial acceleration values are combined into vector acceleration, the vector magnitude (VM) can been seen in Equation 1 (the X, Y, Z is the acceleration of the three axes, N means in unit time interval). Data of acceleration, heart rate and EE are intercepted in 10 and 30s.


(1) $$VM=\frac{\sum_{i=0}^{N-1}\sqrt{x_{i}{}^{2}+y_{i}{}^{2}+y_{i}{}^{2}}\;}{N}$$

Given the difference in EE predictions between exercise and interval, the selection of linear regression and neural network variables was based on previous studies (20, 23). EE is predicted in kcal/10s. Pre-selected variables during exercise included: human morphological index, the unit of 10s corresponds to heart rate during the exercise and the unit of count/10s corresponds to the VM value of each wearing part during the exercise. Since the interval is 10 s, the VM value is the minimum or “0” during the standing interval, and the heart rate during exercise is related to the EE during the interval (31). The pre-selected variable for predicting the intermittent EE includes: human morphological, VM value during 0–10s exercise, VM value during 10–20s exercise, VM value during 0–20s exercise, VM value during entire stage, HR value during 0–10s exercise, HR value during 10–20s exercise, HR value during 0–20s exercise, HR value during entire stage, HR value in Intermittent. Pearson correlation analysis was conducted between pre-selected variables and EE in exercise and interval period, respectively. The absolute value of correlation coefficient (|*r*|) compared the correlation between each variable and EE.

#### Model Development

In order to build LR model, the pre-selected variables of the exercise period and the intermittent period were put into stepwise linear regression, respectively. Then the equations with significance *P*-value of variables < 0.05 were selected. Finally, LR model of exercise period and intermittent period were constructed.

BP neural network is a multi-layer error feedback neural network, which consists of input layer, hidden layer and output layer. Each node represents a neuron, the upper node and the lower node are connected by weight, and the nodes between layers are fully interconnected. Before input variable selection, the temporal characteristics of the four accelerometers were extracted in the non-overlapping windows of 10 and 30 s, and the heart rate (HR) in units of 10 and 30 s was obtained. Input layer variables are selected based on the degree of correlation between EE and the variable, which can include gender, age, weight, height, average heart rate, mean, standard deviation, cova-riance of adjacent windows of data, minimum, maximum, the 10, 25, 50, 75, and 90th percentiles of the vectorial magnitude acceleration. And these variables have been proved to be effective machine learning EE prediction model can be built method (27). In order to improve the speed of convergence, in this study, momentum—learning rate adaptive adjustment algorithm was used to optimize BPNN. The number of hidden nodes was determined by trial and error method. The formula of hidden layer (32) used the Equation 2, “*n*_*i*_” is the number of hidden nodes, “no” is the number of input nodes, and “c” is the number of output nodes. After the number of hidden node is determined, the number of nodes is increased step by step until the number of nodes with the lowest Root mean square error (RMSE) is found. The activation functions of the hidden layer and output layer are determined as logsig logs-type transfer functions. Sigmoid type differentiable function is strictly incremental, with solid theoretical basis, rigorous derivation process, beautiful symmetry of the obtained formula, and strong non-linear fitting ability (33), the formula is the Equation 3. The BPNN model was constructed by the variables of neural network model through Matlab software (R2018b).


(2) $$n=\sqrt{n_{i}+n_{0\;}}+c$$

(3) $$Log\;sig\left(x\right)=\frac{1}{1+e^{-x}}$$

(4) $$RMSE=\sqrt{\frac{1}{n}\sum_{i=1}^{n}{\left({\hat{y}}_{i}-y_{i}\right)}^{2}}$$

### Statistical Analysis

Random grouping was used in which all available data are split into training and testing, at a ratio of 3:1. After grouping, the predicted values were obtained by substituting the test group data into the prediction model constructed by the training. Using Bland-Altman plot to verify the difference between the predicted and measured values, the model is effective when 95% points fall between ± 1.96 SD of the difference between predicted value and measured value. Mean percentage absolute error (MAPE) and RMSE was used to compare the accuracy of prediction between models, the specific formula is the Equations 4 and 5, “ŷ_i_” is the actual measured value, “y_i_” is the predicted value.


(5) $$MAPE=\left(\frac{100}{n}\right)\sum_{i=1}^{n}\left|\frac{\hat{y}i-yi}{yi}\right|$$

## Result

### Correlation Analysis

**Figure 4:**
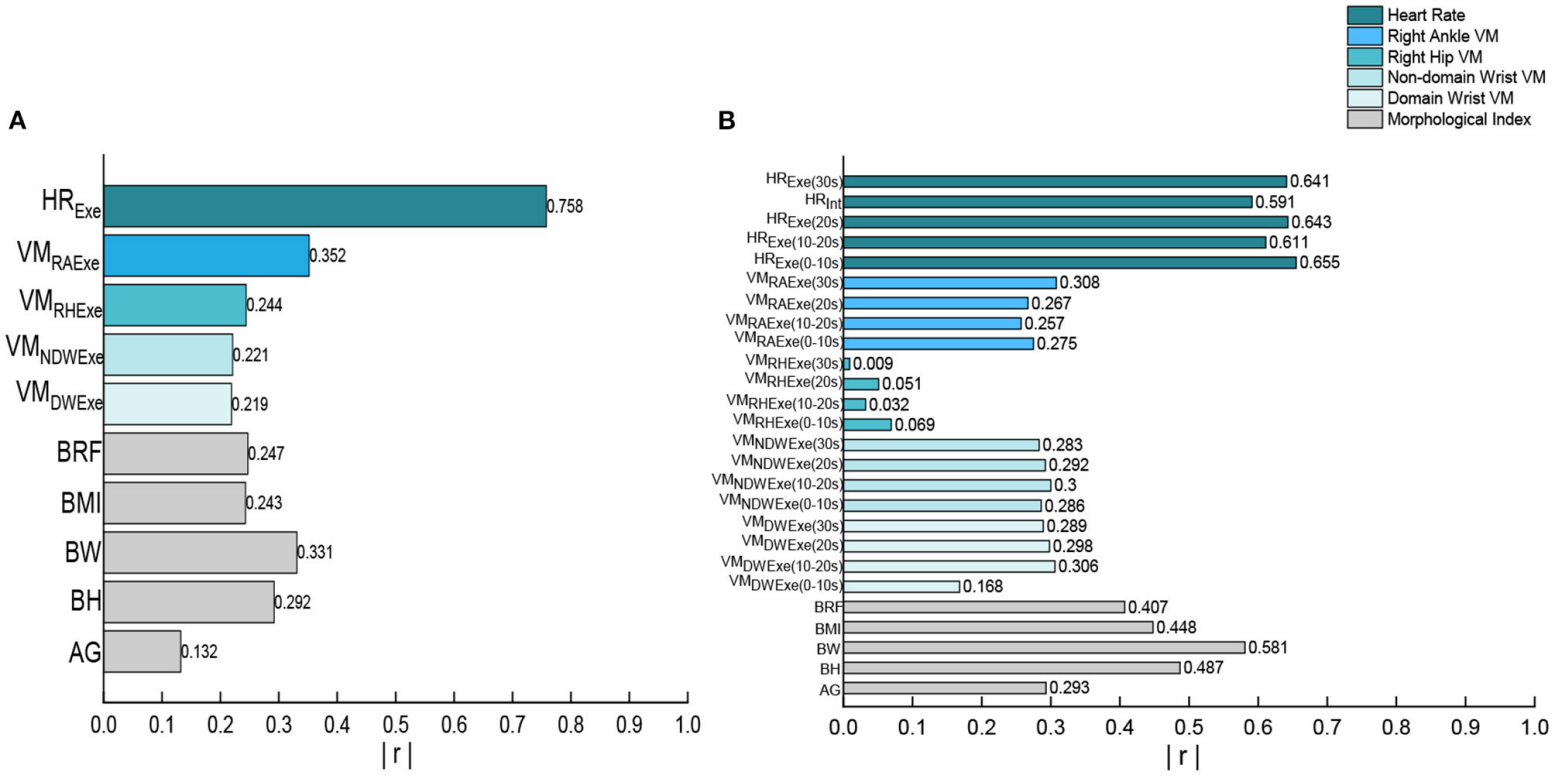
**(A)** Correlation analysis of exercise period; **(B)** Correlation analysis of interval period.

**Figure 5:**
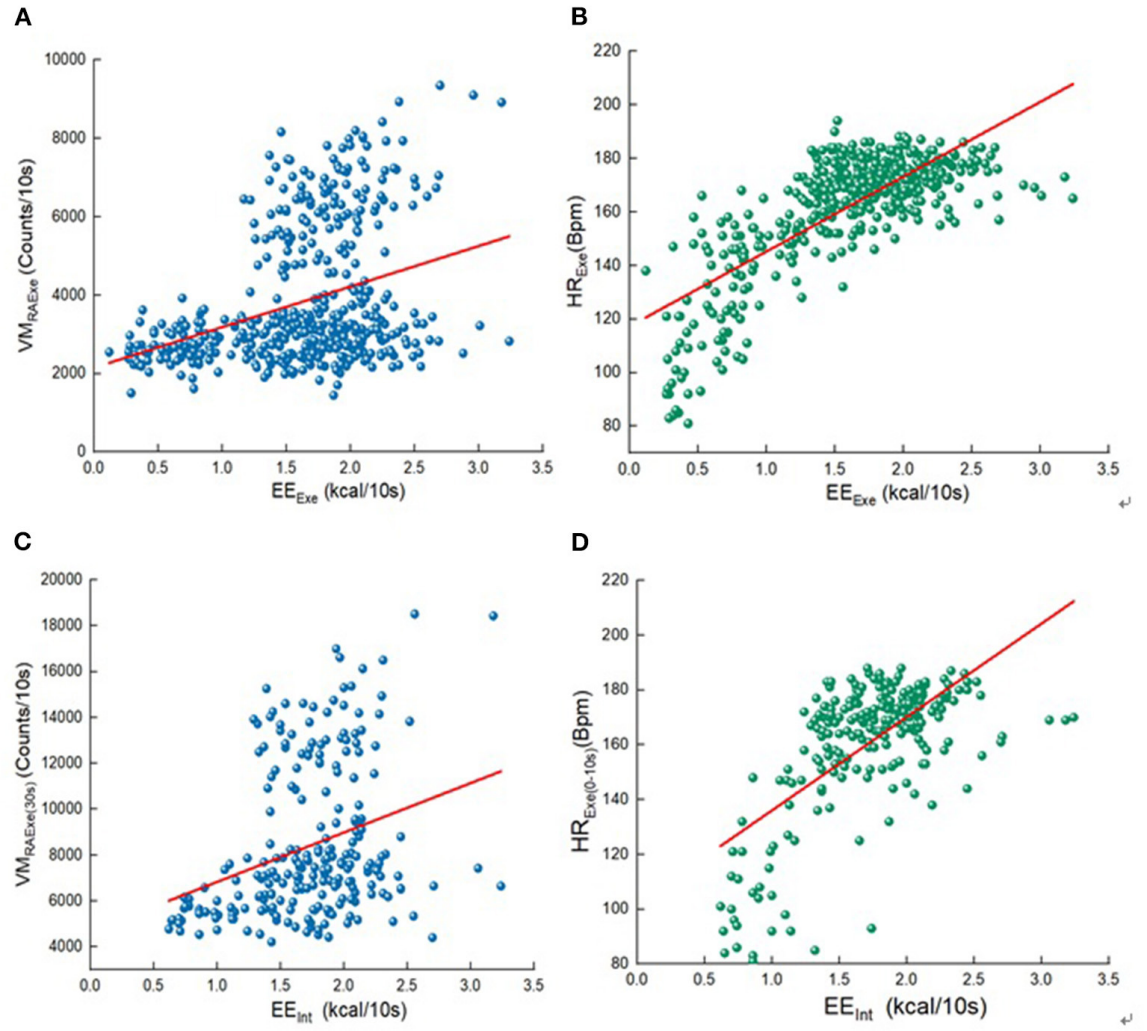
Variables and EE scatter diagram. **(A)** the unit of 10s corresponds to VM during the exercise and EE_Exe_; **(B)** the unit of 10s corresponds to HR during the exercise and EE_Exe_; **(C)** VM value during 30s exercise and EE_Int_; **(D)** HR value during 0–10s exercise and EE_Int_.

According to Pearson correlation analysis, we compared the correlation between each variables and EE (Figure 4). The heart rate band data is the significant correlated with exercise and interval EE. In the various indicators of heart rate, the highest correlation with EE during exercise period is HR value during exercise (*r* = 0.758), the highest correlation with EE during intermittent period is HR value during 0–10s exercise (*r* = 0.655). All the morphologic indicators are significantly correlated with EE, and the highest correlation between anthropometric index and EE in all stages is body weight (*r* = 0.331, *r* = 0.581). Although, the correlation between EE and ACC of multiple wear sites in all stages is significant, the highest correlation variables with EE during exercise and interval period is only moderately correlated, they are right ankle VM value during exercise and right ankle VM value during entire stage (*r* = 0.352, *r* = 0.308). Only the right ankle and hand acceleration are more than 0.300 correlated with EE during the interval. The correlation between the four wearing parts ACC and EE is different in both the exercise and the interval period, and the correlation of ankle is the largest. The measurement indexes of wearable devices with the greatest correlation were analyzed, as shown in Figure 5. There are linear correlation between heart rate measurement indexes and EE in the whole Tabata training, while all the accelerometer indexes are weakly linear correlated with EE.

### Linear Regression

The variables were put into linear stepwise regression, and the LR model was established as shown in Table 2. right ankle VM value during exercise, HR value during exercise and body weight were took as independent variables to build LR model of EE during exercise period. VM value during entire stage, HR value during 0–10s exercise and body weight were took as independent variables to build LR model of EE during interval period. The significance *P*-values of these independent variables in the LR model are all < 0.05, and they are all valid variables. The *r*^2^ value of the EE prediction model in exercise period is 0.710, and the *r*^2^ value of the EE prediction model in interval period is 0.730. The significance of the model is < 0.05, indicating that the model is valid.

**Table 2:** EE prediction model of Tabata linear regression.

**Use conditions**	**Model**	***r* ^2^**
Exercise	EE_Exe_ = 0.000044*VM_RAExe_ + 0.193*HR_Exe_ + 0.23*BW-3.05	0.71
Interval	EE_Int_ = 0.000011*VM_RA(30s)_ + 0.0116*HR_Exe(0−10s)_ + 0.030*BW-1.99	0.73

### BP Neural Network

**Figure 6:**
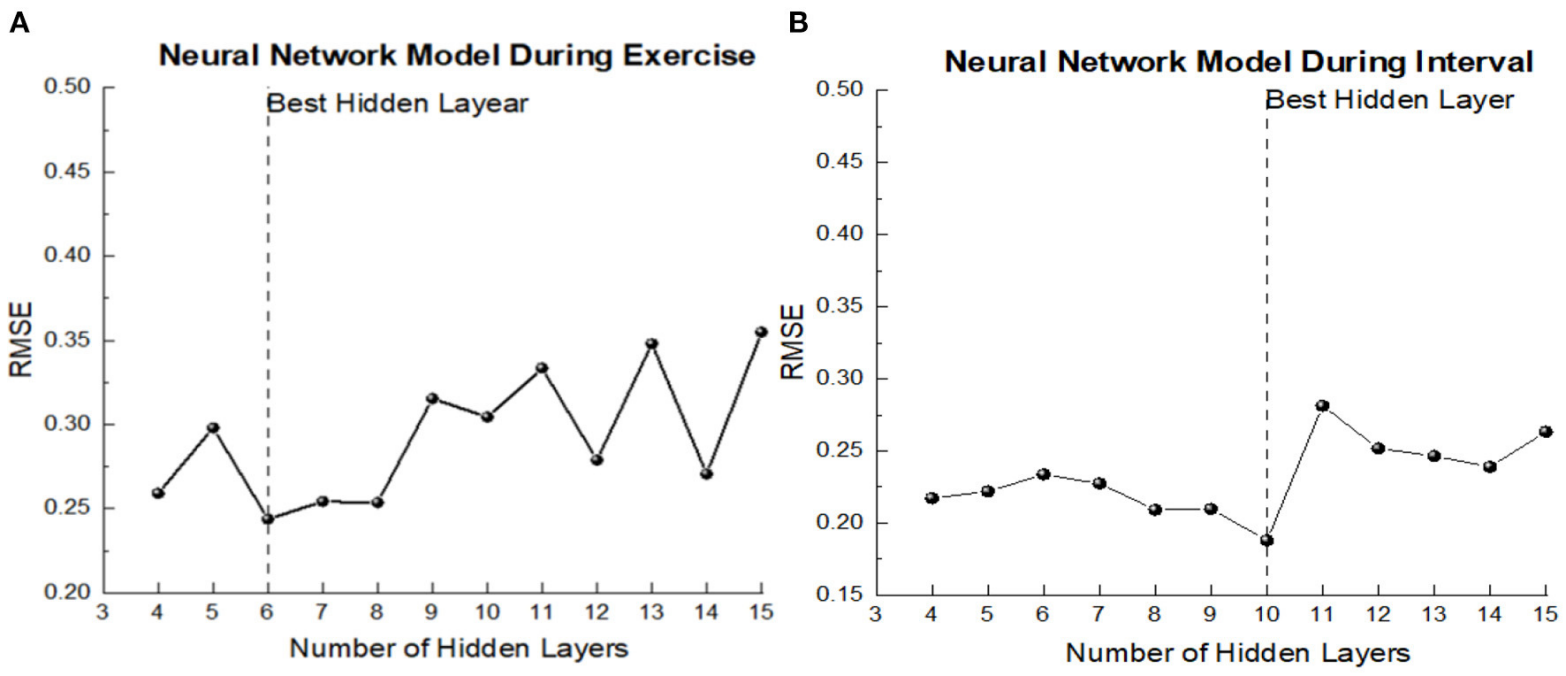
The prediction error of model of each hidden node. **(A)** Exercise period; **(B)** Interval period.

Based on the correlation between the acceleration data of each position and EE during all stages, this study decided to take the right ankle VM value during exercise as the input variable of the BPNN model during the exercise period, and the right ankle VM value during entire stage was input as the input variable of the BPNN model in intermittent period. HR in exercise period was selected as the input variable value of the neural network model during the exercise period, and HR value during 0–10s exercise was taken as the input variable of the BPNN model during the exercise period. Height, weight and gender were input into the two models as the anthropometric index and basic information of participants. After using trial and error method, we determined 4–15 hidden layers as an attempt, and finally got the hidden layers of the neural network as shown in Figure 6. The number of hidden layers of model in exercise period was 6, and that of model in intermittent period was 10.

**Figure 7:**
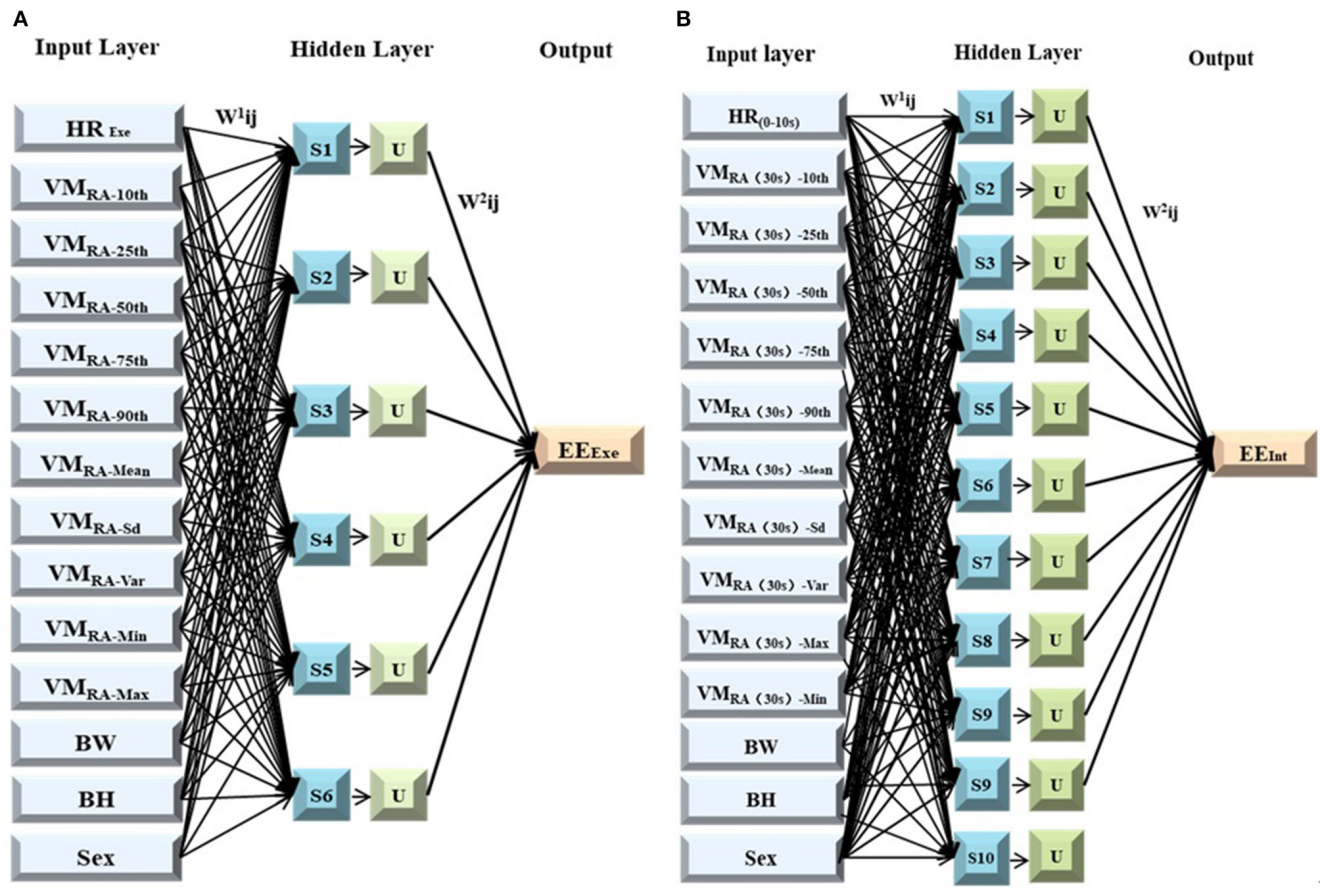
Model structure. **(A)** BP neural network during exercise; **(B)** BP neural network during exercise.

Finally, the neural network model was determined as 14-6-1 in the exercise period and 14-10-1 in the interval period. The upper limit of network iteration of neural network model is 5,000 times, initial learning rate is 0.05, momentum constant is 0.9 and error rate is 0.001, as shown in Figure 7. “w1” and “w2” in turn represent input and sum weight vectors, “Si” means the sum of the input layers in the implicit cell, “U” indicates that the input activation function is hidden. The training group was substituted into the model for training and BPNN was finally obtained. After calculation, the time complexity of BPNN model during exercise and interval period is 20139840 and 27643680, respectively. The *r*^2^ value of the exercise model is 0.813, the *r*^2^ value of the intermittent model is 0.816, which indicates the model is valid.

### Predictive Accuracy of Models

**Figure 8:**
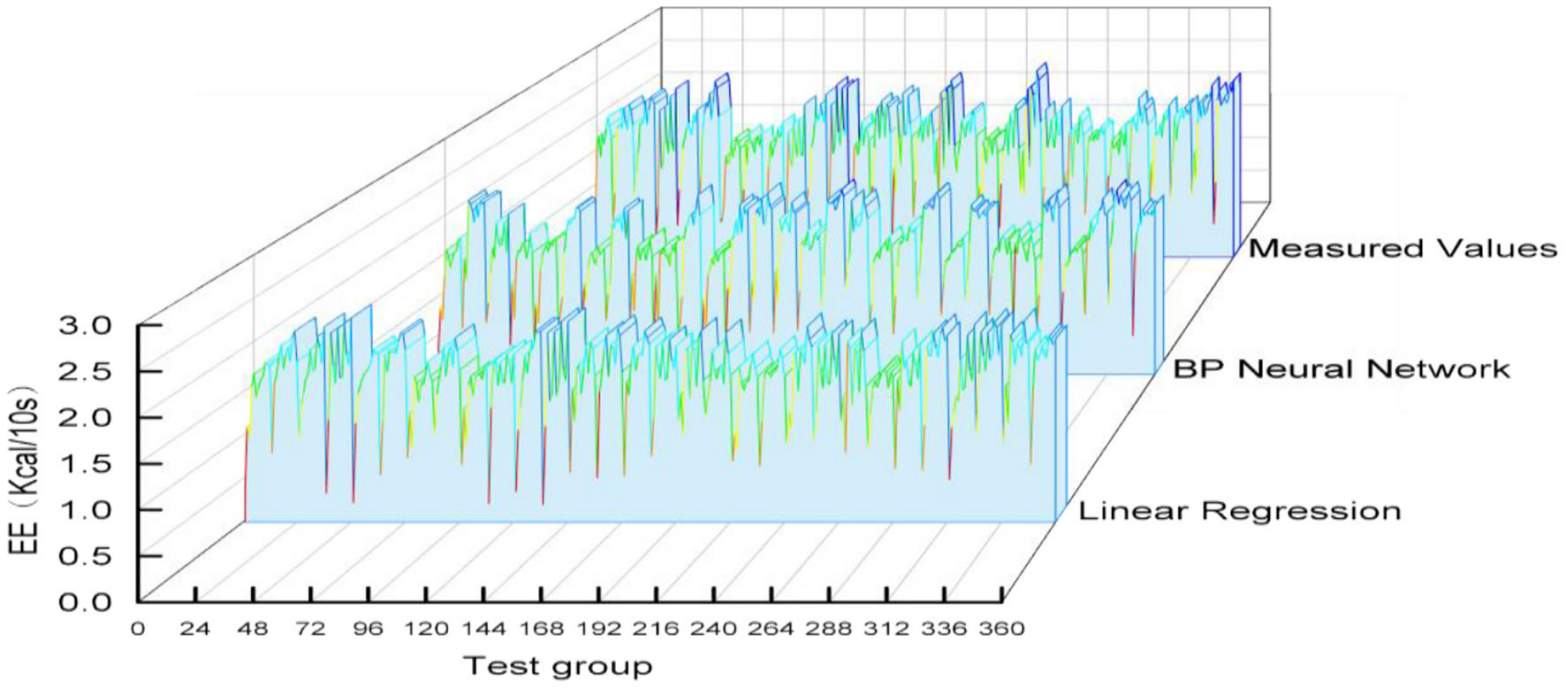
Predicted values and Measured values.

The variable data of the test group was substituted into each prediction model and the predicted values of each model were obtained. Then the predicted EE values and measured EE values of 15 subjects in the validation group were intercepted in 10-s increments, each subject will have 24 predicted values. The whole verification group was substituted into each model to obtain 24^*^15 predicted values. The forecast and measured graph was presented in Figure 8.

**Figure 9:**
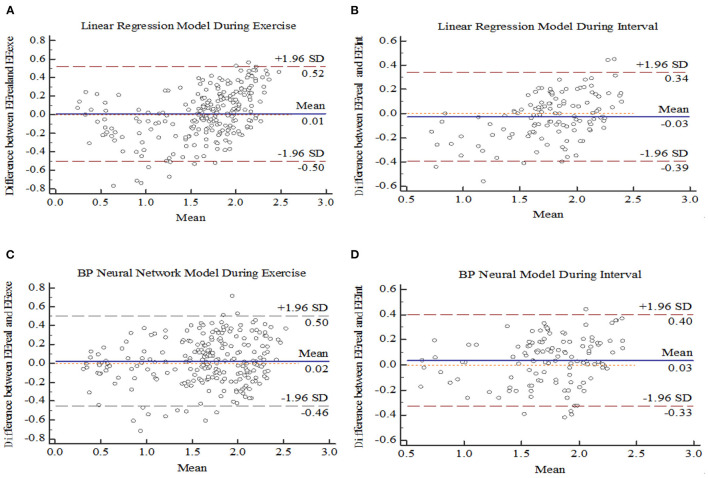
Bland Altman plot for models. **(A)** Linear regression during Exercise. **(B)** Linear regression during interval. **(C)** BP neural network during Exercise **(D)** BP neural network during interval.

Bland-Altman Plot statistical method with MedCalc software was used to analyze the consistency of model prediction effect, as shown in Figure 7. The difference between the measured EE value of K4b2 and the predicted EE value of each model was used as the Y-axis, and the mean value of the predicted EE value of each model and the measured K4b2 was used as the X-axis. Figure 9 showed that 95% of the scatter points of the two prediction models were within the range of ± 1.96 SD during the exercise period and interval period, they all have good consistency.

**Figure 10:**
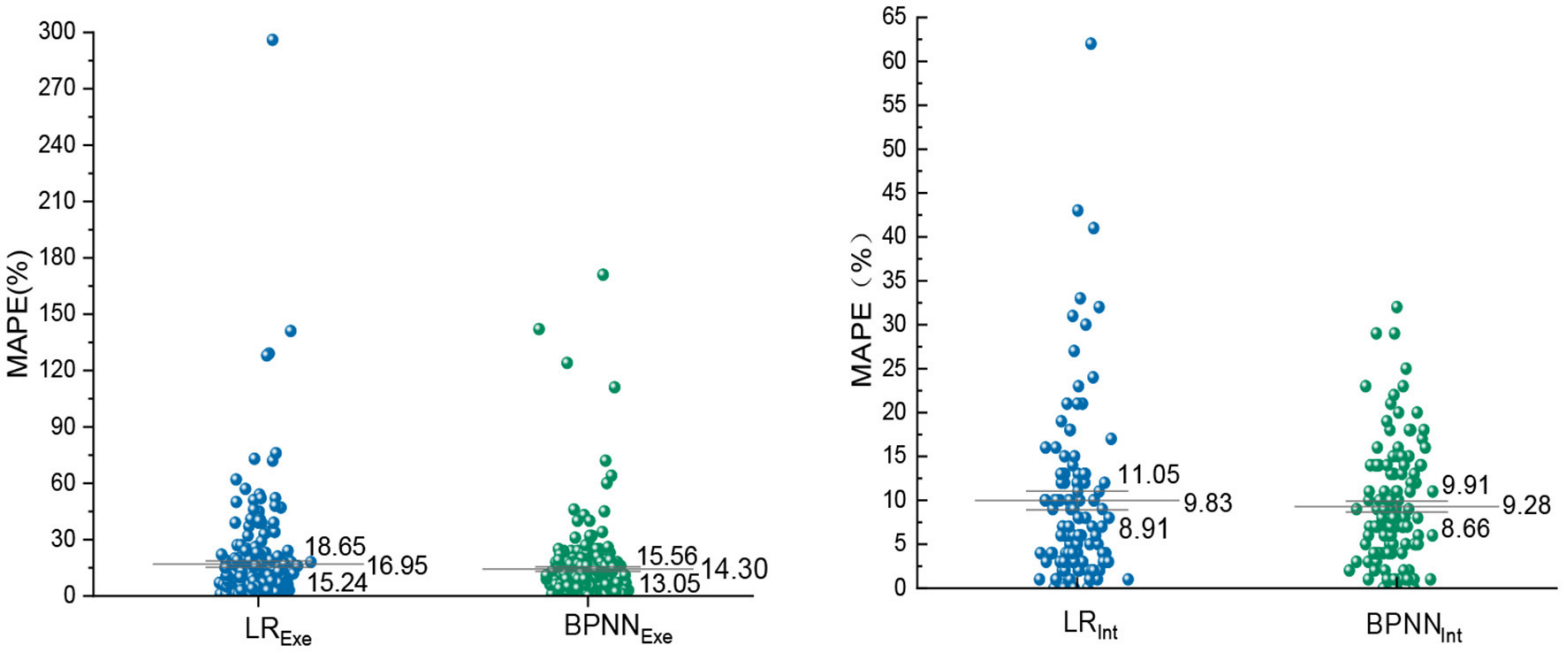
Prediction error of EE during exercise and interval period.

The predicted and measured values of the validation group were substituted into the MAPE formula for calculation, and the MAPE of the two models in the exercise period and the intermittent period were compared. The MAPE of LR model is 16.95% in EE prediction during exercise period, which is 2.65% higher than BPNN model. In the intermittent EE prediction, the MAPE of BPNN model is 9.28%, which is lower than LR model. At the same time, the error dispersion degree of LR model is larger, and its standard error of MAPE is 1.70% and 1.22%, which proves that the stability of LR prediction effect is lower than that of BPNN (Figure 10).

**Figure 11:**
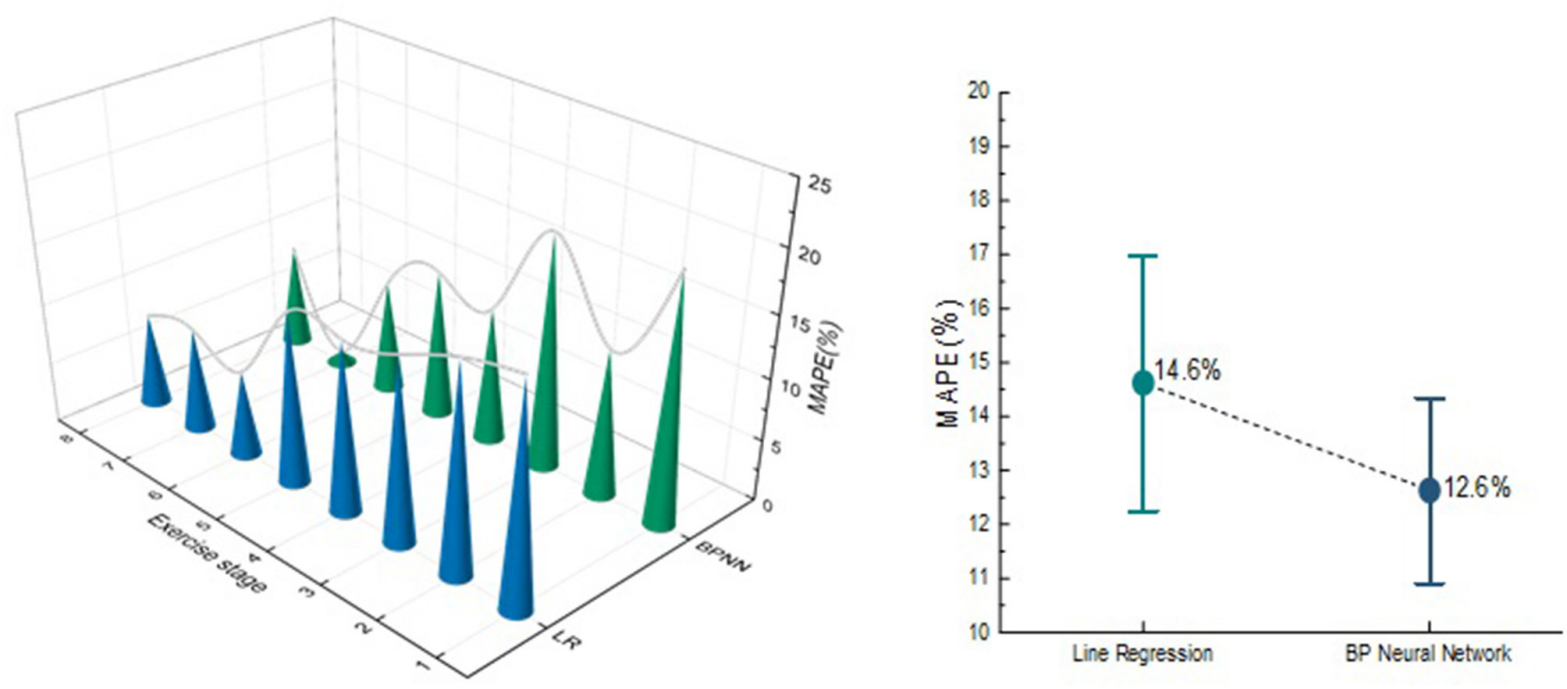
MAPE comparison of the EE of total exercise.

In order to compare the prediction effect of the Tabata training during each movement stage, the two models were compared in each exercise stage, as shown in Figure 11. In the LR model, the MAPE of three exercise stages is >15%, while the BPNN model has two. In addition, the MAPE and RMSE predicted by tow EE model in entire Tabata training was calculated. The MAPE of BPNN model was 12.6%, which is 2% lower than that of LR model, and the RMSE of LR model was 0.238, which is 0.011 higher than that of BPNN model. Both LR and BPNN models can effectively predict the EE of Tabata, but BPNN model has better prediction performance than the LR model regardless of the comparison of each part or the whole movement.

## Discussion

In this study, the correlation of pre-selected variables was analyzed. The highest correlation with the EE in the exercise and the intermittent period is HR, which basically shows a linear relationship with the EE. The linear correlation between HR and EE in high-intensity exercise can also be confirmed in Tabata training (16). Bazuelo et al. (30) has shown that intermittent heart rate is related to intermittent EE. But this study found that intermittent EE not only correlate with intermittent heart rate, but also with exercise heart rate, the highest correlation with intermittent EE was the HR value during 0–10s exercise, and the correlation was basically linear. Meanwhile Dugas et al. show the similar result (31), the exercise they tested was set-up exercise (45s-exercise, 15s-interval), the significant correlation of intermittent EE is exercise HR 1 min before the interval. Due to a temporal dissociation be-tween HR and VO2 (EE) during intermittent-type activity, the relationship between resting heart rate and oxygen uptake was not strong during partial exercise intervals (34), Therefore, the HR value during 0–10s exercise was selected as the input variable in this study. At present, there are few studies on the correlation between exercise heart rate and intermittent energy, and the correlation between other exercises and intermittent energy expenditure is not clear, which needs to be verified in the future.

Throughout the Tabata training, the ACC has a moderate correlation with EE, showing a weak linear correlation with EE, and the ankle ACC is the highest correlation among all the wearing parts. Tabata tested in this study included high-intensity and complex movements. In this exercise mode, the ACC values of each part of the body are vary a lot and do not show periodic changes, and the different parts of the body show different physical activity. This is similar to the study by Morris et al. (22), who validated smart devices with built-in accelerometers worn on the hip and wrist. The high intensity intermittent motion was carried out, the prediction efficiency of the acceleration values of the two wear parts is not good after substituting into the existing linear model. Although some studies suggest that accelerometers worn on the hip can better predict EE, most of these studies tested continuous periodic motion (35). Some studies have also suggested that ankle is the most suitable wearing part for sports with high speed and intensity, because the acceleration signal vector of human motion changes with different postures (36). Therefore, the ankle acceleration can be used as a prediction variable in EE prediction in this study.

Some studies have also shown the correlation between EE and anthropometric indexes (37). In this study, the correlation between body weight and EE was the highest among anthropometric indexes. Therefore, body weight is also considered a variable of EE prediction.

After the correlation analysis of the pre-selected variables, the LR model was constructed with the heart rate, the right ankle VM value and body weight as independent variables through stepwise regression of the predictive variables. Referring to input variables selected in past studies, BPNN is constructed by combining relevant variables significantly related to EE with subject characteristics information.

In terms of model establishment, Bland-Altman method is used to test that LR and BPNN model are consistent. The feasibility of predicting EE with heart rate combined accelerometer was confirmed, which was consistent with the results of previous studies by Brage and Crouter (38, 39). A recent study evaluated the effectiveness of accelerometers in predicting the EE of High Intensity Functional interval Training (HIFT), which is similar to the Tabata model. But, the effect of accelerometer alone on the EE prediction by linear regression model is not ideal, and the MAPE of energy expenditure in the whole movement process is more than 15%, and the errors in predicting EE during exercise and interval period have not been specifically evaluated (22). In accordance to the statement made by Lee et al. (40) that the reasonable error of energy expenditure prediction is 10–15%. Compared with the recent study, the overall prediction errors of the two ACC-HR models constructed in this study are both lower than 15%, which has improved the prediction effect in EE prediction of high intensity intermittent exercise. By comparing LR and BPNN model, the MAPE of BPNN model is higher than 15% in two stages, while the MAPE in LR model is higher than 15% in three stages. In addition, the RMSE and MAPE predicted by BPNN in the whole exercise process were lower than those predicted by LR model (0.227 and 12.6%), which proved that BPNN model based on machine learning algorithm in the era of big data could provide exercisers with more accurate prediction of EE than LR, which was similar with the result of Montoya et al. (15).

## Limitation

Firstly, Limitations of test contents, this study selected classic and representative Tabata exercise pattern and movement for testing. Although the model cannot predict the EE of all Tabata exercises, it can be used to predict EE in the classical pattern. Future research can further enrich the testing of Tabata exercises and modes, so as to improve the accuracy of Tabata EE prediction. In addition, Limitations of the applicability, the subjects selected were 45 young adults. Therefore, the generalization of the models established in this study to other age group further research and confirmation.

## Conclusion and Future Work

In this paper, a linear regression and neural network model were constructed to predict the EE of Tabata training using accelerometer combined with heart rate data. The two models have good consistency by bland-Alterman test, and the prediction performance is evaluated by MAPE and RMSE, which proves that the error of back propagate neural network is lower than linear regression. Overall, the empirical results showed that: (i) the effectiveness of acceleration combined with heart rate data in predicting Tabata exercise EE. (ii) Back propagate neural network model constructed by acceleration combined with heart rate has higher prediction accuracy than linear regression model, reflecting the feasibility of machine learning algorithm in predicting EE of complex intermittent exercise. The ACC-HR Back propagate neural network model provides exercisers with a portable and more accurate tool for predicting Tabata EE, so as to help exercisers adjust their physical activities and set up their exercise plans, thus improving their exercise benefits. In the future, more novel and useful machine learning algorithms will be used to predict the EE of high intensity interval exercise. In addition, this study will test more Tabata exercise patterns and movements to more fully and accurately predict Tabata exercise EE.

## Data Availability Statement

The original contributions presented in the study are included in the article/supplementary material, further inquiries can be directed to the corresponding author/s.

## Ethics Statement

The studies involving human participants were reviewed and approved by Chengcheng Cai, Sport Laboratory, College of Physical Education, Sichuan Normal University. The patients/participants provided their written informed consent to participate in this study.

## Author Contributions

YY participated in research concepts, design, subject data collection, manuscript writing, and chart making. QC contributed to the research concept and made critical revisions to the research design and manuscript. All authors contributed to the paper, the manuscript was constantly revised, and the authors unanimously agreed on the version submitted.

## Funding

The work was supported by the Physical Education Institute of Sichuan Normal University, which provided the venue for the testing.

## Conflict of Interest

The authors declare that the research was conducted in the absence of any commercial or financial relationships that could be construed as a potential conflict of interest.

## Publisher's Note

All claims expressed in this article are solely those of the authors and do not necessarily represent those of their affiliated organizations, or those of the publisher, the editors and the reviewers. Any product that may be evaluated in this article, or claim that may be made by its manufacturer, is not guaranteed or endorsed by the publisher.

## Abbreviations

Tabata: Tabata interval training
HR: heart rate
VM: vector magnitude
BPNN: back propagate neural network
LR: line regression
HIFT: high Intensity functional training.
